# Predictive value of creatine kinase MB for contrast-induced acute kidney injury among myocardial infarction patients

**DOI:** 10.1186/s12872-021-02155-7

**Published:** 2021-07-13

**Authors:** Wen Wei, Lingyu Zhang, Yunhan Zhang, Ronghui Tang, Miao Zhao, Zhidong Huang, Jin Liu, Danyuan Xu, Yibo He, Bo Wang, Haozhang Huang, Qiang Li, Mengfei Lin, Yong Liu, Kaihong Chen, Shiqun Chen

**Affiliations:** 1 Department of Cardiology, Guangdong Provincial Key Laboratory of Coronary Heart Disease Prevention, Guangdong Cardiovascular Institute, Guangdong Provincial People’s Hospital, Guangdong Academy of Medical Sciences, Guangzhou, 510080 China; 2 Department of Endocrinology, Longyan First Affiliated Hospital of Fujian Medical University, Longyan, 364000 China; 3 The Second School of Clinical Medicine, Southern Medical University, Guangzhou, 510515 China; 4 Department of Cardiology, Maoming People’s Hospital, Maoming, 525000 China; 5 Kunming Medical University, Kunming, 650500 China; 6 Department of Ultrasound Imaging, Yunnan Fuwai Cardiovascular Hospital, Kunming, 650500 China; 7 Guangdong Provincial People’s Hospital, School of Medicine, South China University of Technology, Guangzhou, 510100 China; 8 Department of Cardiology, Longyan First Affiliated Hospital of Fujian Medical University, Longyan, 364000 China

**Keywords:** Creatine kinase-MB, Contrast-induced acute kidney injury, Predictive, Myocardial infarction

## Abstract

**Background:**

Predictive value of creatine kinase MB (CK-MB) for contrast-induced acute kidney injury (CI-AKI) among myocardial infarction (MI) patients has rarely been reported. We aim to evaluate the predictive value of CK-MB for CI-AKI among MI patients.

**Methods:**

Totally, 1131 MI patients were included from the REduction of rIsk for Contrast-Induced Nephropathy (REICIN) study. The peak CK-MB before coronary angiography (CAG) was chosen. The study population was divided into two groups by log-transformed CK-MB cut-off point. The association between CK-MB and CI-AKI was tested by multivariable logistic regression. CK-MB was integrated with Age, creatinine and ejection fraction (ACEF) score and Mehran risk score (MRS) to evaluate the additive value of CK-MB. The integrated models were validated internally by the bootstrap method and externally by the PREdictive Value of COntrast voluMe to creatinine Clearance Ratio (PRECOMIN) study data set.

**Results:**

Overall, 62(5.48%) patients developed CI-AKI, patients with CK-MB point > 4.7 displayed a higher incidence of CI-AKI than those without (11.9% vs. 4.0%, *p* < 0.001). CK-MB point > 4.7 was independently associated with CI-AKI (adjusted OR: 3.40, 95% CI: 1.93–5.98, *p* < 0.001). The additions of CK-MB to ACEF score, Mehran score A and Mehran score B resulted in increases in C-statistics, which ranged from 0.680 to 0.733 (*p* = 0.046), 0.694 to 0.727 (*p* = 0.091), 0.704 to 0.734 (*p* = 0.102), respectively. Internal validation also showed increases in C-statistics, and external validation performed well in discrimination and calibration.

**Conclusions:**

Preprocedural peak CK-MB was a predictor of CI-AKI among MI patients.

## Introduction

Contrast-induced acute kidney injury (CI-AKI) is the third most common cause of hospital-acquired renal failure, with an incidence of 11% [1], and is associated with poor short- and long-term outcomes [2–5]. As an important and readily available cardiac biomarker, creatine kinase MB (CK-MB) has long been used in the diagnosis of acute myocardial infarction (AMI) because of its good cost-performance ratio and simplicity [6]. CK-MB has also been indicated to improve clinical risk prediction of postoperative acute kidney injury (AKI) among patients undergoing cardiac surgery [7]. In a study of 257 patients, Katarzyna ZR et al. found that increased CK-MB and Red Cell Distribution Width (RDW) are associated with higher risk of CI-AKI among patients with AMI [8]. However, few studies have investigated the independent predictive value of CK-MB in CI-AKI among myocardial infarction (MI) patients.

Therefore, we aim to evaluate the independent predictive utility of CK-MB to CI-AKI risk and determine whether CK-MB can add predictive information to the traditional risk models for determining CI-AKI among MI patients undergoing coronary angiography (CAG) or percutaneous coronary intervention (PCI).

## Methods

### Data sources and study population

This study included 1131 MI patients from the multicenter prospective REduction of rIsk for Contrast-Induced Nephropathy (REICIN) study from January 2013 to June 2016 (trial registration: ClinicalTrials.gov NCT01402232). Only adult patients (≥ 18 years of age; referred to CAG or PCI) with providing written informed consent were studied. This study was conducted in accordance with the Declaration of Helsinki and was approved by the Research Ethics Committee of Guangdong Provincial People’s Hospital, Guangdong Academy of Medical Sciences (No. GDREC2012141H).Follow-up data was monitored and recorded by trained nurses and research assistants through outpatient interviews and telephones.

### Variables and study endpoint

Biochemistry data CK-MB was evaluated on admission, at 3-h intervals in the first 24 h, and daily in the first 3 days following admission. The peak CK-MB before CAG or PCI was chosen. Immunosuppressive method was used to determine the activity of CK-MB and was applied to each sub-center. Due to its non-normal distribution, the CK-MB variable was log-transformed. Receiver operating characteristic (ROC) curve was used to determine the cut-off point of optimal prognostic performance. Then the CK-MB was for the next analysis as a categorical variable based on the cut-off point.

Serum creatinine (Scr) concentration was measured at admission and within 24, 48 and 72 h after CAG or PCI. Other biochemical indicators were evaluated on admission. The echocardiography examination was used to evaluate the left ventricular ejection fraction (LVEF).

The primary endpoint was CI-AKI_0350_, defined as an increase in the Scr by over 0.3 mg/dL or over 50% from baseline within the first 48 h after the CAG [9]. The secondary endpoint was 3-year all-cause mortality.

### ACEF score and Mehran risk score (MRS)

Age, creatinine and ejection fraction (ACEF) score was calculated by evaluating age, Scr and LVEF [10]. MRS was calculated by evaluating the presence of hypotension, congestive heart failure(CHF), anemia, and diabetes mellitus(DM), the use of intra-aortic balloon pump(IABP), age > 75 years, the amount of contrast medium, and the basal renal function. There are two types of MRS: Mehran score A using Scr as a criterion for renal function, and Mehran score B using estimated glomerular filtration rate (eGFR) [11].

### Validation cohort

The PREdictive Value of COntrast voluMe to creatinine Clearance Ratio (PRECOMIN, trial registration:ClinicalTrials.gov NCT01400295) study [12] was a prospective single-center observational study that reviewed all consecutive patients (n = 3369) undergoing CAG and/or PCI between January 2010 and October 2012 according to the institutional protocol. The PRECOMIN study included 1312 MI patients, of which 511 samples had no data deletion. Among 511 patients, 58 (11.35%) patients fulfilled the diagnostic criteria for CI-AKI_0350_.

### Statistical analyses

Continuous variables were expressed as mean (standard deviation [SD]) or medians interquartile range (IQRs), and discrete variables were expressed as frequency counts and percentages. The differences in variables among groups were evaluated by the t-test or chi-square test. The association between CK-MB and CI-AKI was tested by univariable and multivariable logistic regression. And then, CK-MB was integrated with ACEF score and MRS to compare the predictive power of before and after addition. The performances were evaluated based on discrimination and calibration. Discrimination was evaluated with the ROC curve and expressed by the C-statistic. The C-statistics were compared by the Delong test. We also compared the models using the continuous net reclassification index (NRI) and integrated discrimination and improvement (IDI). The calibration of these models was described by the Hosmer–Lemeshow test.

To evaluate the stability of the integrated models, these models were validated internally using 1000 bootstrap samples and externally validated in the PRECOMIN study data set. We calculated an optimal bootstrap-corrected C-statistic as described by Riley et al. by fitting the prediction model in each of the 1000 bootstrap samples [13]. External validation was furthermore assessed by both discrimination and calibration.

All analyses were performed with R software (version 4.0.3; R Foundation for Statistical Computing, Vienna, Austria). A two-sided *p*-value < 0.05 indicated significance for all analyses.

## Result

### Baseline clinical characteristics and outcomes

From January 2013 to June 2016, a total of 1131 consecutive MI patients who underwent CAG or PCI were included. The mean age was 60.87 ± 12.05 years, and 932 patients (82.40%) were males. 62 (5.48%) patients fulfilled the diagnostic criteria for CI-AKI_0350_. The cut-off point of log-transformed CK-MB for the best predictive value of CI-AKI was 4.7. Therefore, patients were divided into two groups based on the cut-off point: 920 (81.34%) patients with log-transformed CK-MB ≤ 4.7, and 211(18.66%) patients with log-transformed CK-MB > 4.7. All of the baseline clinical characteristics of the patients are shown in Table 1. Overall, there were 607(53.67%) patients with chronic kidney disease (CKD), 295(26.08%) patients with DM and 562 (49.69%) patients with hypertension.

**Table 1:** Comparison of clinical characteristics between patients with and without elevated CK-MB

Characteristic	Overall (n = 1131)	log(CK-MB) ≤ 4.70(n = 920)	log(CK-MB) > 4.70 (n = 211)	*p*-value
*Demographic characteristics*				
Age (years)	60.87 ± 12.05	60.90 ± 12.00	60.71 ± 12.28	0.834
Male gender	932 (82.40%)	761 (82.72%)	171 (81.04%)	0.634
Weight (kg)	65.74 ± 10.84	65.71 ± 10.96	65.89 ± 10.31	0.842
BMI (kg/m^2^)	23.91 ± 3.21	23.91 ± 3.24	23.93 ± 3.06	0.958
*Medical history and clinical condition*				
CI-AKI	62 (5.48%)	37 (4.02%)	25 (11.85%)	< 0.001
Smoking history	542 (47.92%)	424 (46.09%)	118 (55.92%)	0.012
DM	295 (26.08%)	243 (26.41%)	52 (24.64%)	0.659
Hypertension	562 (49.69%)	471 (51.20%)	91 (43.13%)	0.042
Hyperlipidemia	139 (12.29%)	106 (11.52%)	33 (15.64%)	0.127
CKD	607 (53.67%)	496 (53.91%)	111 (52.61%)	0.790
CHF	460 (40.67%)	368 (40.00%)	92 (43.60%)	0.377
Anterior infarction	399 (35.28%)	291 (31.63%)	108 (51.18%)	< 0.001
Hypotension	86 (7.60%)	59 (6.41%)	27 (12.80%)	0.003
Anemia	321 (28.38%)	275 (29.89%)	46 (21.80%)	0.023
IABP	42 (3.71%)	28 (3.04%)	14 (6.64%)	0.022
LVEF (%)	55.17 ± 11.34	55.91 ± 11.37	52.02 ± 10.69	< 0.001
*Procedure*				
Emergent PCI	976 (86.30%)	782 (85.00%)	194 (91.94%)	0.011
*Laboratory examination*				
GLU(mmol/L)	8.16 ± 3.89	8.03 ± 3.88	8.71 ± 3.91	0.022
Hb(g/L)	128.05 ± 20.46	128.73 ± 20.21	125.05 ± 21.34	0.020
hct	40.62 ± 5.87	40.39 ± 5.89	41.64 ± 5.68	0.006
Scr(mg/dl)	95.37 ± 40.66	96.20 ± 42.43	91.72 ± 31.62	0.148
CCR(ng/ml)	73.70 ± 28.88	73.36 ± 28.86	75.18 ± 28.98	0.410
eGFR(ml/min/1.73mm^2^)	80.98 ± 26.73	80.69 ± 26.89	82.26 ± 26.04	0.441
BUN(mmol/L)	5.00 [3.90, 6.50]	4.91 [3.90, 6.48]	5.20 [4.03, 6.58]	0.143
HDL-C(mmol/L)	1.00 ± 0.28	0.98 ± 0.27	1.08 ± 0.33	< 0.001
ALB(g/L)	36.10 ± 5.15	36.07 ± 5.12	36.23 ± 5.31	0.712
*Treatment during hospitalization*				
ACEI/ARB	145 (12.82%)	133 (14.46%)	12 (5.69%)	0.001
Beta-blockers	611 (54.02%)	532 (57.83%)	79 (37.44%)	< 0.001
CCB	89 (7.87%)	84 (9.13%)	5 (2.37%)	0.002
Statins	912 (80.64%)	764 (83.04%)	148 (70.14%)	< 0.001
Hypoglycemic drugs	125 (11.05%)	115 (12.50%)	10 (4.74%)	0.002
Diuretic	261 (23.08%)	201 (21.85%)	60 (28.44%)	0.050
Contrast dose(ml)	113.80 ± 50.15	112.14 ± 50.05	121.02 ± 50.08	0.021

*CK-MB* creatine kinase isoenzymes/creatine kinase-MB, *BMI* body mass index, *CI-AKI* contrast-induced acute kidney injury, *DM* diabetes mellitus, *CKD* chronic kidney disease, *CHF* congestive heart failure, *IABP* intra-aortic ballon pump, *LVEF* left ventricular ejection fraction, *PCI* percutaneous coronary intervention, *GLU* blood glucose, *Hb* hemoglobin, *hct* hematocrit, *Scr* serum creatinine, *CCR* Creatinine Clearance Rate, *eGFR* estimated glomerular filtrationrate, *BUN* blood urea nitrogen, *HDL-C* high density lipoprotein cholesterol, *ALB* albumin, *ACEI/ARB* angiotensin-converting enzyme inhibitor/angiotensin receptor blocker, *CCB* calcium channel blocker

Compared with patients with log-transformed CK-MB ≤ 4.7, patients with log-transformed CK-MB > 4.7 demonstrated lower LVEF (52 ± 11vs. 56 ± 11, *p* < 0.001) and higher random plasma glucose (8.7 ± 3.9 vs. 8.0 ± 3.9 mmol/l, *p* = 0.022). These patients also showed higher incidences of hypotension on the day of admission (12.8% vs. 6.4%, *p* = 0.003), anterior infarction (51.2% vs. 31.6%, *p* < 0.001) and IABP (6.6% vs. 3.0%, *p* = 0.022). Nonetheless, there were no significant differences in the incidences of CKD, DM and CHF between the two groups (*p* > 0.05) (Table 1).

### Predictive value of CK-MB in CI-AKI

Compared with patients with log-transformed CK-MB ≤ 4.7, patients with log-transformed CK-MB > 4.7 displayed a significantly greater incidence of CI-AKI (11.9% vs. 4.0%, *p* < 0.001, Table 1). The area under the CK-MB curve in relation to CI-AKI was 0.625 (95% confidence interval [CI]: 0.550 to 0.701), and a Hosmer–Lemeshow χ^2^ statistic of 11.37 (*p* = 0.182). Univariate logistic regression analysis indicated that log-transformed CK-MB > 4.7 was significantly correlated with CI-AKI (odds ratio [OR]: 3.21, 95% CI: 1.89–5.46, *p* < 0.001, Table2). In multivariable logistic regression, log-transformed CK-MB > 4.7 had an independent association with CI-AKI (adjusted OR: 3.40, 95% CI: 1.93–5.98, *p* < 0.001, Table 2). Other variables associated with CI-AKI included age, Scr and LVEF. Table 2 depicts the performance of each covariate in logistic regression analysis.

**Table 2:** Univariate and multivariate logistic regression analysis of CI-AKI_0305_

	Univariate		Multivariate	
	OR (95%CI)	*p*-value	OR(95%CI)	*p*-value
Age(years)	1.05 (1.02,1.07)	< 0.001	1.04 (1.02,1.07)	0.001
Scr	1.01 (1.01,1.01)	< 0.001	1.01 (1.00,1.01)	< 0.001
Contrast dose(ml)	1.00 (1.00,1.01)	0.419	1.00 (1.00,1.01)	0.384
DM	1.37 (0.79,2.38)	0.256	1.28 (0.72,2.28)	0.404
LVEF (%)	0.96 (0.94,0.98)	< 0.001	0.98 (0.96,1.00)	0.036
CK-MB	3.21 (1.89,5.46)	< 0.001	3.40 (1.93,5.98)	< 0.001

*CI-AKI* contrast-induced acute kidney injury, *Scr* serum creatinine, *DM* diabetes mellitus, *LVEF* left ventricular ejection fraction, *CK-MB* creatine kinase isoenzymes/creatine kinase-MB

### Discrimination and calibration analysis of CK-MB added to models

**Fig. 1:**
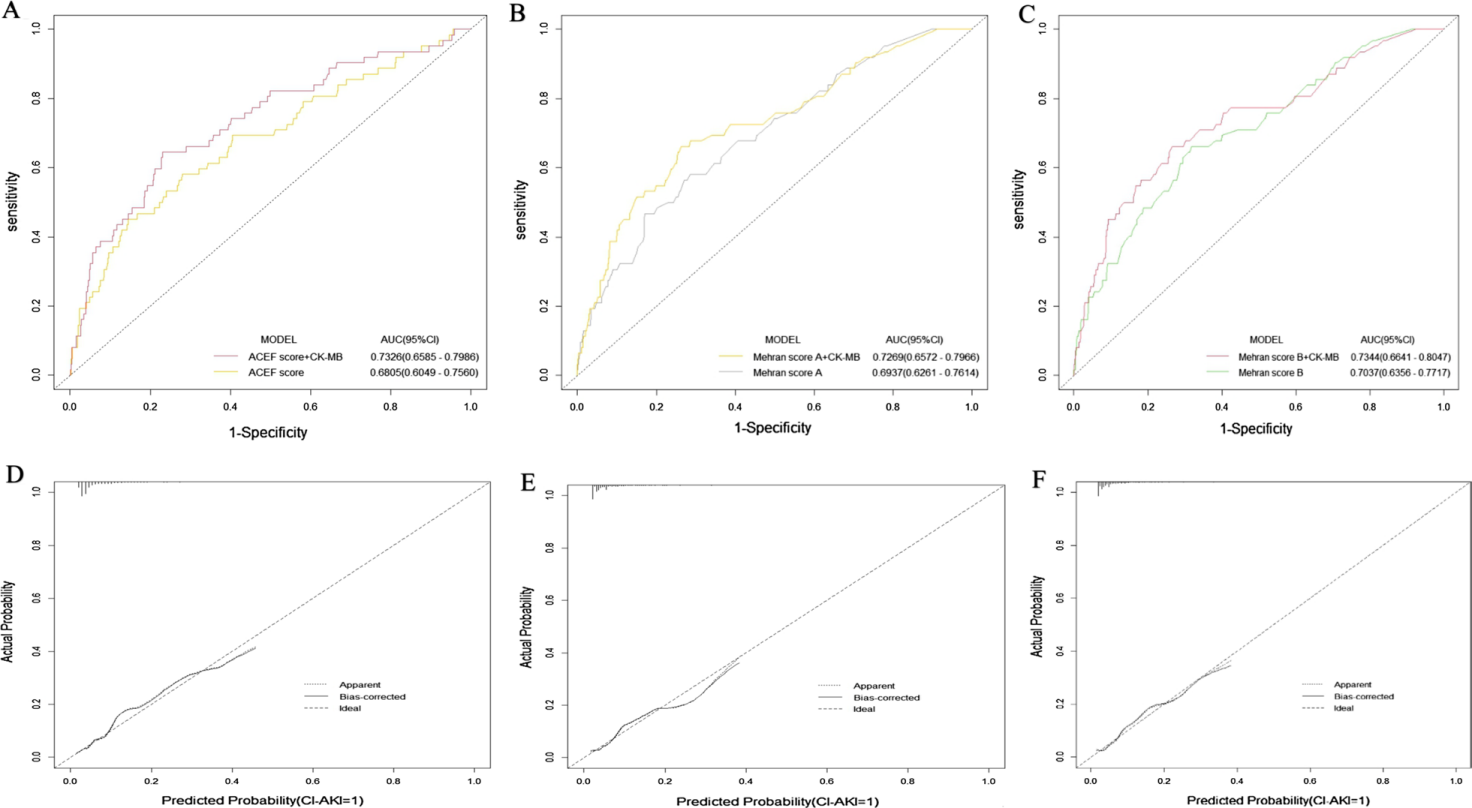
The performance of CK-MB before and after adding ACEF score and MRS in REICIN study data set. **a** The ROC curves of ACEF score and ACEF score + CK-MB for CI-AKI. **b** The ROC curves of Mehran score A and Mehran score A + CK-MB for CI-AKI. **c** The ROC curves of Mehran score B and Mehran score B + CK-MB for CI-AKI. **d** The bootstrap curve of ACEF score + CK-MB integrated model for CI-AKI. **e** The bootstrap curve of Mehran score A + CK-MB integrated model for CI-AKI. **f** The bootstrap curve of Mehran score B + CK-MB integrated model for CI-AKI. *AUC* area under the receiver operating characteristic curve, *ROC* receiver operator characteristic, *CK-MB* creatine kinase isoenzymes/creatine kinase-MB, *MRS* Mehran risk score, *ACEF* Age, creatinine and ejection fraction, *REICIN* REduction of rIsk for Contrast-Induced Nephropathy

**Fig. 2:**
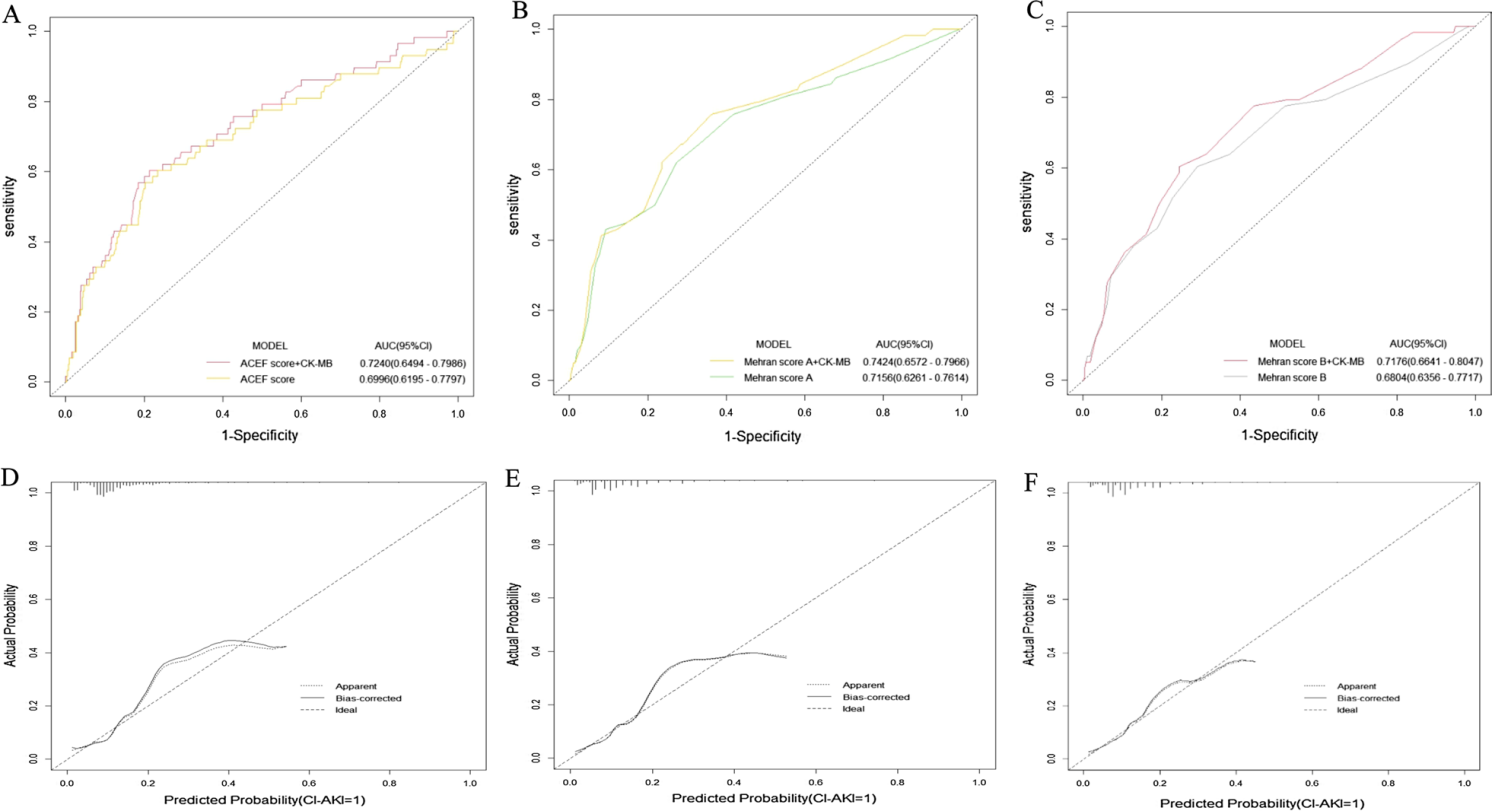
The performance of CK-MB before and after adding ACEF score and MRS in PRECOMIN study data set. **a** The ROC curves of ACEF score and ACEF score + CK-MB for CI-AKI. **b** The ROC curves of Mehran score A and Mehran score A + CK-MB for CI-AKI. **c** The ROC curves of Mehran score B and Mehran score B + CK-MB for CI-AKI. **d** The bootstrap curve of ACEF score + CK-MB integrated model for CI-AKI. **e** The bootstrap curve of Mehran score A + CK-MB integrated model for CI-AKI. **f** The bootstrap curve of Mehran score B + CK-MB integrated model for CI-AKI. *AUC* area under the receiver operating characteristic curve, *ROC* receiver operator characteristic, *CK-MB* creatine kinase isoenzymes/creatine kinase-MB, *MRS* Mehran risk score, *ACEF* Age, creatinine and ejection fraction, *PRECOMIN* PREdictive Value of COntrast voluMe to creatinine Clearance Ratio

The addition of the categorical variable log-transformed CK-MB to ACEF score, Mehran score A and Mehran score B did contribute to increase in C-statistics, which ranged from 0.680 to 0.733 (*p* = 0.046), 0.694 to 0.727 (*p* = 0.091), 0.704 to 0.734 (*p* = 0.102), respectively (Fig. 1, Table 3). And the CK-MB added significant discriminative value to the traditional models when evaluated by NRI and IDI (Table 3). These models embodied a good calibration for CI-AKI based on the Hosmer–Lemeshow test (Table 3). By internal bootstrap validation, the bootstrap-corrected C-statistics ranged from 0.680 to 0.731, 0.695 to 0.725, 0.702 to 0.731, respectively (Table 3). Additionally, the external validation data set had similar good performance in discrimination and calibration (Table 4, Fig. 2). The external validation shown the addition of CK-MB to ACEF score, Mehran score A and Mehran score B did contribute to increase in C-statistics, which ranged from 0.700 to 0.724 (*p* = 0.059), 0.716 to 0.742 (*p* = 0.024), 0.680 to 0.718 (*p* = 0.002), respectively (Table 4).

**Table 3:** Discrimination and calibration analysis of CK-MB added to models in REICIN study data set

	C-statistics	*p*-value	NRI	*p*-value	IDI	p-value	Hosmer and Lemeshow test	AIC	Bootstrap-corrected C-statistics
ACEF + CK-MB	0.733	0.046	0.459	< 0.001	0.018	0.017	χ^2^ = 8.75, *p* = 0.364	440.24	0.731
ACEF score	0.680						χ^2^ = 3.39, *p* = 0.907	452.91	0.680
Mehran A + CK-MB	0.727	0.091	0.459	< 0.001	0.017	0.021	χ^2^ = 7.02, *p* = 0.534	440.33	0.725
Mehran score A	0.694						χ^2^ = 5.09, *p* = 0.748	451.33	0.695
Mehran B + CK-MB	0.734	0.102	0.459	< 0.001	0.018	0.020	χ^2^ = 9.31, *p* = 0.317	436.60	0.731
Mehran score B	0.704						χ^2^ = 7.07, *p* = 0.529	447.95	0.702

*CK-MB* creatine kinase isoenzymes/creatine kinase-MB, *ACEF* Age, creatinine and ejection fraction, *REICIN* REduction of rIsk for Contrast-Induced Nephropathy

**Table 4:** Discrimination and calibration analysis of CK-MB added to models in PRECOMIN study data set

	C-statistics	p-value	NRI	*p*-value	IDI	*p*-value	Hosmer and Lemeshow test	AIC	Bootstrap-corrected C-statistics
ACEF + CK-MB	0.724	0.059	0.236	< 0.001	0.015	< 0.001	χ^2^ = 8.93, *p* = 0.348	440.24	0.723
ACEF score	0.700						χ^2^ = 5.62, *p* = 0.690	340.16	0.699
Mehran A + CK-MB	0.742	0.024	0.236	< 0.001	0.011	0.027	χ^2^ = 3.90, *p* = 0.866	329.31	0.737
Mehran score A	0.716						χ^2^ = 6.40, *p* = 0.602	334.14	0.714
Mehran B + CK-MB	0.718	0.002	0.236	< 0.001	0.012	0.026	χ^2^ = 8.40, *p* = 0.395	336.98	0.714
Mehran score B	0.680						χ^2^ = 5.31, *p* = 0.725	342.46	0.680

*CK-MB* creatine kinase isoenzymes/creatine kinase-MB, *ACEF* Age, creatinine and ejection fraction, *PRECOMIN* PREdictive Value of COntrast voluMe to creatinine Clearance Ratio

## Discussion

In this multicenter, prospective study of MI patients who underwent CAG or PCI, we found that preprocedural peak CK-MB was an independent predictor of CI-AKI. The risk of CI-AKI was over 3.4-fold among patients with log-transformed CK-MB > 4.7 than those without. The addition of the variable CK-MB to either ACEF score or MRS did result in increasing CI-AKI risk demonstrated by C-statistics. And the CK-MB added significant discriminative value to the traditional models when assessed by NRI and IDI. All the results suggested CK-MB plays a key role in the clinical predictive value for CI-AKI among MI patients undergoing CAG or PCI.

In concordance with our results, one previous study recognized increased CK-MB and RDW levels were significantly associated with CI-AKI among AMI patients. AMI patients with CK-MB > 55 U/L were 1.2-fold more likely to develop CI-AKI than those without [8]. However, that single-center retrospective study, with a relatively small amount of AMI patients, did not research the independent predictive value of CK-MB for CI-AKI and the correlation between CK-MB and long-term prognosis. Our multicenter prospective study, including 1131 nonselective MI population, described both the independent predictive value of CK-MB for CI-AKI and the association of CK-MB with long-term prognosis.

Luis et.al. indicated that an independent association was not enough to establish the usefulness of a biomarker. They suggested inserting a new variable into a traditional risk scoring tool, and then comparing the performance of the traditional predictive model with that of an alternative model [14]. The ACEF score, a traditional risk model, has already been considered as a risk scoring of CI-AKI among patients undergoing primary PCI for a user-friendly clinical parameter by a quick preprocedural prediction of CI-AKI [10, 15, 16]. The MRS was another forecasting tool widely used to stratify the probability of developing CI-AKI after PCI [11]. After integrating variable CK-MB with the traditional risk models, we found the C-statistics significantly increased.

The mechanisms underlying the prediction of CI-AKI by CK-MB may be related to hemodynamic instability. The elevation of preprocedural CK-MB, indicating the extent of myocardial necrosis, was closely related to the occurrence of cardiogenic shock and heart failure [17–19]. A consequent decrease in cardiac output led to a decline of renal perfusion as well as renal ischemia, resulting in AKI ultimately. Although troponin T and troponin I were more sensitive than CK-MB in detecting minor myocardial damage, measurement of CK-MB may be used to provide a facile clinical estimation of the infarct size [20]. Furthermore, troponin T and troponin I have not been uniformly used in low- and middle- income countries in the past clinical practice, which may result in bias due to different detection quality. Moreover, there are defects in Roche's high quality tests, which are more expensive than tests of CK-MB.

Our study also found that patients with log-transformed CK-MB > 4.7 had lower LVEF than those without. A decrease of LVEF indicated the loss of contractility due to acute ischemia or myocardial necrosis [21, 22]. Several studies have indicated that worsened LVEF was a predictor of CI-AKI [23–26]. In accordance with the previous study [23, 27, 28], our study demonstrated that the age and basal creatinine were independently correlated with CI-AKI. Cinar T et al. also indicated that the age, creatinine and ejection fraction score correlated with ST-elevation myocardial infarction-related cardiogenic shock [29]. CK-MB might be a promising and timely tool for predicting CI-AKI among such MI patients. Therefore, regular monitoring, preventive strategies, and even priority treatment should be given to patients with log-transformed CK-MB > 4.7 for a well renal outcome in MI patients.

### Limitation

First, the definition of CI-AKI was diverse. We adopted a definition of CI-AKI_0350_ based on the increase in Scr, and used both baseline and postprocedural values, which only gave a moderately accurate evaluation of renal function. However, the definition of CI-AKI in our research was commonly cited in previous studies. Second, the log-transformed CK-MB may be complex in clinical applications. This defect will limit to generalize our results. Third, since there were 855 missing data of troponin T and 822 missing data of troponin I in our study, it is hard to detect the predictive value of troponin for CI-AKI. Fourth, the single center in PRECOMIN study is one of the centers in REICIN study, but the subjects in the two studies were enrolled at different periods.

## Conclusion

The present study might be the first to report that the preprocedural peak CK-MB is a powerful indicator of CI-AKI among MI patients. The application of such a readily available biomarker may help clinicians to make a judgment on the CI-AKI risk of the MI patients. Log-transformed CK-MB > 4.7 may be an optimal target for patients to receive therapeutic measures to prevent CI-AKI.

## Data Availability

Data relevant to this study are available from the corresponding authors upon reasonable request.

## Abbreviations

CK-MB: Creatine kinase MB
CI-AKI: Contrast-induced acute kidney injury
AKI: Acute kidney injury
MI: Myocardial infarction
AMI: Acute myocardial infarction
CAG: Coronary angiography
PCI: Percutaneous coronary intervention
RDW: Red Cell Distribution Width
ACEF: Age, creatinine and ejection fraction
MRS: Mehran risk score
LVEF: Left ventricular ejection fraction
CHF: Congestive heart failure
DM: Diabetes mellitus
IABP: Intra-aortic balloon pump
CKD: Chronic kidney disease
GLU: Blood glucose
Hb: Hemoglobin
hct: Hematocrit
Scr: Serum creatinine
CCR: Creatinine Clearance Rate
eGFR: Estimated glomerular filtrationrate
BUN: Blood urea nitrogen
HDL-C: High density lipoprotein cholesterol
ALB: Albumin
ACEI: Angiotensin-converting enzyme inhibitor
ARB: Angiotensin receptor blocker
CCB: Calcium channel blocker
ROC: Receiver operating characteristic
NRI: Net reclassification index
IDI: Integrated discrimination and improvement
REICIN: REduction of rIsk for Contrast-Induced Nephropathy
PRECOMIN: PREdictive Value of COntrast voluMe to creatinine Clearance Ratio
